# Impact of abdominal compression on heart and stomach motion for stereotactic arrhythmia radioablation

**DOI:** 10.1002/acm2.14346

**Published:** 2024-04-25

**Authors:** Daniel David Cecchi, Nicolas Paul Ploquin, Salman Faruqi, Hali Morrison

**Affiliations:** ^1^ Department of Physics and Astronomy University of Calgary Calgary Alberta Canada; ^2^ Department of Medical Physics Tom Baker Cancer Centre Calgary Alberta Canada; ^3^ Department of Oncology Division of Medical Physics University of Calgary Calgary Alberta Canada; ^4^ Department of Radiation Oncology Tom Baker Cancer Centre Calgary Alberta Canada; ^5^ Department of Oncology Division of Radiation Oncology University of Calgary Calgary Alberta Canada

**Keywords:** immobilization, stereotactic radiotherapy, ventricular radioablation

## Abstract

**Purpose:**

To evaluate the effectiveness of abdominal compression (AC) as a respiratory motion management method for the heart and stomach during stereotactic arrhythmia radioablation (STAR).

**Methods:**

4D computed tomography (4DCT) scans of patients imaged with AC or without AC (free‐breathing: FB) were obtained from ventricular‐tachycardia (VT) (*n* = 3), lung cancer (*n* = 18), and liver cancer (*n* = 18) patients. Patients treated for VT were imaged both FB and with AC. Lung and liver patients were imaged once with FB or with AC, respectively. The heart, left ventricle (LV), LV components (LVCs), and stomach were contoured on each phase of the 4DCTs. Centre of mass (COM) translations in the left/right (LR), ant/post (AP), and sup/inf (SI) directions were measured for each structure. Minimum distances between LVCs and the stomach over the respiratory cycle were also measured on each 4DCT phase. Mann‐Whitney U‐tests were performed between AC and FB datasets with a significance of *α* = 0.05.

**Results:**

No statistical difference (all *p* values were >0.05) was found in COM translations between FB and AC patient datasets for all contoured cardiac structures. A reduction in COM translation with AC relative to FB was patient, direction, and structure specific for the three VT patients. A significant decrease in the AP range of motion of the stomach was observed under AC compared to FB. No statistical difference was found between minimum distances to the stomach and LVCs between FB and AC.

**Conclusions:**

AC was not a consistent motion management method for STAR, nor does not uniformly affect the separation distance between LVCs and the stomach. If AC is employed in future STAR protocols, the motion of the target volume and its relative distance to the stomach should be compared on two 4DCTs: one while the patient is FB and one under AC.

## 1 INTRODUCTION

Stereotactic arrhythmia radioablation (STAR) is a novel treatment option using radiotherapy (RT) for refractory ventricular tachycardia (VT),^1^ an arrhythmia originating in the lower chambers of the heart. The current standard of care for VT is catheter ablation (CA)—an invasive procedure utilizing radiofrequency heat to target the cells in the myocardial walls causing the arrhythmic events^2^; VT episode recurrence can occur for up to 49% of patients with myocardial infarction post‐CA.^3^ STAR is an alternative treatment option for patients with recurring arrhythmic episodes post‐CA or patients not otherwise eligible for CA. In recent STAR studies, the target is prescribed a dose of 25 Gy to the 70%−80% isodose line, following the findings of prior pre‐clinical studies on swine and current published literature.^1^, ^4^, ^5^, ^6^, ^7^ Some recent publications on STAR have shown excellent results in reducing VT burden and arrhythmic episode recurrence in their respective patient cohorts by up to 99.9%.^1^, ^8^, ^9^ However, other studies have shown little VT burden reduction or VT recurrence when treating with STAR,^7^, ^10^ demonstrating the need for further investigation on the possible patient or treatment‐specific factors affecting treatment success.

Questions remain about the proper management of the target volume's intrafraction motion for STAR patients, which is subject to both the cardiac and respiratory cycle.^11^, ^12^ A common approach to account for target motion during treatment is creating an internal target volume (ITV) expansion applied to the gross target volume (GTV). However, this increases the total amount of irradiated tissue. Other motion management techniques, such as respiratory gating and breath hold methods, have been assessed for their potential to reduce STAR target motion during beam‐on time. They have been shown to be effective methods for some patients.^13^, ^14^ Another management option that has been used in select STAR studies is abdominal compression (AC).^8^, ^15^ The function of AC is to limit lung expansion and diaphragm contraction during inhalation and is an effective respiratory motion management device for liver and some lower‐lobe lung tumors.^16^, ^17^ In current STAR studies, AC has been employed or evaluated as part of the treatment workflow^8^, ^18^; however, it has yet to be studied for its effect on STAR target respiratory‐induced motion compared to free‐breathing (FB; without AC).

In addition to the intrafraction target motion, avoiding organs at risk (OARs) surrounding the target volume is also essential in radiotherapy to limit normal tissue toxicity, which could affect patients’ quality of life, and to avoid severe radiation toxicity complications. An OAR of particular concern for STAR is the stomach, given its proximity to the heart and potentially severe complications from excess radiation exposure during high‐dose single‐fraction radiotherapy. To mitigate this risk, a reduction in target dose coverage is considered to meet the dose constraints of the stomach or the stomach planning organ at risk volume (PRV) to ensure the dose received by the stomach is minimized.^19^

Inter‐ and intra‐fraction OAR motion, such as that present with the stomach during radiotherapy, may affect target coverage for treatments in the abdomen and increase normal tissue toxicity.^20^, ^21^, ^22^ Reducing abdominal organ motion is possible with AC (such as for the liver); however, it has yet to be evaluated for its effect on stomach motion relative to STAR target locations. Gerard et al.^18^ notably did not employ AC for their single‐patient case study of ventricular radioablation because the stomach was brought closer to the PTV under AC. It is unknown whether AC causes this effect for each patient and to what extent.

A recent review by Stevens et al.^23^ discussed the reported cardiac‐respiratory motion of patients within existing STAR studies. The authors report that the variability in cardiac‐respiratory motion between patients and studies demonstrates a significant uncertainty in prescribing the optimal motion‐management technique. The work presented here evaluates the effect of AC on the motion of the heart, LV, and possible STAR target locations, as well as the impact of AC on stomach motion and stomach‐treatment margin overlap. This study aims to determine whether AC is an effective method to reduce heart target motion during ventricular radioablation and if the distance between the target and stomach tissue is affected by AC.

## 2 METHODS AND MATERIALS

4DCT scans of previously treated lung and liver SBRT patients imaged FB or with AC, respectively, were collected and imported into MIM^©^ (MIM Software Inc, Cleveland, OH). The AC bridge and compression paddle (CDR Freedom System™) were placed on the patient's abdomen to minimize diaphragmatic motion. The compression level was set to the maximum tolerable level that each patient indicated they could withstand for the duration of treatment, as per local clinical protocol. Patient data was acquired with local ethics approval ([Ethics Number Redacted]). All patients included in the analysis had a 4DCT scan with a 2 mm slice thickness, which matched the local STAR protocol, adequate scan quality with minimal or negligible image artifacts, and the whole stomach and heart were contained in the scan for proper contouring. In addition, three patients treated with STAR following a local protocol were also imported into MIM^©^ for analysis. The STAR patients were scanned with and without AC (using the same compression protocol as liver SBRT patients). In total, 42 4DCT scans were obtained: 18 lung (FB), 18 liver (AC), and 3 VT patients (FB and AC). All patients were imaged using a Philips Big Bore CT Simulator (Philips N.V., Amsterdam, Netherlands). The total patient dataset demographic and the VT patient demographic and treatment characteristics are shown in Tables 1 and 2, respectively. The target locations of the three VT patients are described according to the 17‐segment map defined by the American Heart Association.^24^

**TABLE 1:** FB and AC patient demographic included in the study.

	FB	AC
Patient Demographic	*N* = 21% (*n*)	*N* = 21% (*n*)
Mean Age [years]	74	68
Age Range [years]	58‐92	44‐85
Female	57 (12)	24 (5)
Male	43 (9)	76 (16)

**TABLE 2:** STAR patient treatment characteristics.

Patient and treatment characteristics	VT Patient 1	VT Patient 2	VT Patient 3
Gender, Age	Male, 72	Male, 62	Male, 72
Prescription Dose – 80% isodose line	25 Gy	25 Gy	25 Gy
Energy	6X‐FFF	6X‐FFF	6X‐FFF
Target Location	1, 2, 7, 8	3‐5, 10, 11, 15, 16	2, 3, 5, 6, 8, 9, 11, 12
ITV PTV	2 mm Isotropic 3 mm Isotropic	None 3 mm Isotropic	2 mm Isotropic 3 mm Isotropic
*Volume [cc]*			
GTV	22.60	44.66	11.84
ITV	40.29	N/A	23.98
PTV	73.59	103.64	52.09
Immobilization	AC + VacQfix™	AC + VacQfix™	AC + VacQfix™

Abbreviation: FFF, Flattening‐Filter‐Free.

The heart, LV, and LV components (LVC) (anterior, apical, inferior, lateral, and septal) were contoured following the Duane et al. heart atlas^25^ on each phase of the 4DCTs. Limbus AI^©^ (2022 Limbus AI Inc, Saskatchewan, Canada)^26^ was used to contour the stomach on each phase of the 4DCT. Stomach contours were individually reviewed and corrected as needed. Ten RT‐structure files in DICOM format corresponding to the 10 4DCT phases were extracted from Limbus, which were then re‐imported into MIM^©^ and applied to the corresponding phase for each patient. Relative center of mass (COM) translations in the left/right (LR), ant/post (AP), and sup/inf (SI) directions for all contoured structures were calculated by MIM^©^. The 0% phase of the 4DCT was taken as the reference phase for all relative COM translations. A positive COM translation is defined towards the patient's left, posterior, and superior direction relative to the location of the defined structure on the reference phase. Python^©^ (v3.9.12, 2022 Python Software Foundation, https://www.python.org/, Beaverton, OR) scripts were used to process and analyze all data. Mann‐Whitney U‐tests (MW‐test) were performed between all FB and AC datasets with a significance level of *α* = 0.05. For all analysis methods, a detailed comparison was performed between FB and AC data from the three VT patients, in addition to comparing the total FB and AC datasets (*N* = 42) due to them being imaged under both FB and AC conditions.

### 2.1 Relative COM Translations

Two methods were performed for COM translation comparisons between FB and AC patients:

1. Maximum magnitude COM translation for all contoured structures:
$$\begin{array}{ccc} & & \max\{\sqrt{{\mathrm{LR}-\mathrm{COM}}_{i}^{2}+{\mathrm{AP}-\mathrm{COM}}_{i}^{2}+{\mathrm{SI}-\mathrm{COM}}_{i}^{2}};\\ & & \;i\in [0\%,\text{…},\;90\%]\},\forall \;\text{Patients} \end{array}$$
2. ROM (range of motion) in the LR, AP, and SI direction of the LVCs and stomach:
$$\begin{array}{ccc} {\text{ROM}}_{X} & = & \max\left\{T\right\}-\min\left\{T\right\};\;T=\{X-\mathrm{CO}M_{i};\\ i & \in & [0\%,\text{…},\;90\%]\};X\in [\mathrm{LR},\mathrm{AP},\mathrm{SI}],\forall \;\text{Patients} \end{array}$$

The purpose of incorporating both analyses is to evaluate total structure motion and any possible directional response of the structure to AC. ROM from the exported COM translations represents the structure's maximum translation in each direction, assuming a rigid, non‐deforming contour. For the three VT patients, simplified anisotropic ITV margins were applied to the clinical GTVs to evaluate the effect of AC on treatment volumes; the applied ITV margins were defined as one‐half the ROM in each direction assuming symmetrical motion about the 50% respiratory phase; this method is purely to compare FB to AC ROM and does not necessarily represent clinical ITVs. For the case where the target volume spanned multiple LVCs, the larger of the ROMs was taken to be applied onto the GTV.

### 2.2 Relative Distance Between LVCs to Stomach

The exported RT‐structure files were stored as 3D points corresponding to the contoured structure. Using Scipy© (Scipy, 2022, https://scipy.org), a math package for Python^©^, the minimum distances over each 4DCT phase between each LVC and the stomach were calculated. The following variables defined the sets used for analysis: the minimum distance overall respiratory phases and whether the minimum distance fell within a 3 or 5 mm margin to the LVCs. These two margin values were chosen based on STAR treatment margins in the literature.^1^, ^7^, ^8^, ^27^, ^28^, ^29^ FB and AC sets were then compared with the MW‐test with significance *α* = 0.05 to evaluate the effect of AC compared to FB. A regression analysis was performed between the magnitude COM translation of the LVCs (averaged over the respiratory‐cycle) and the minimum distance from the LVCs to the stomach over the entire respiratory‐cycle. The obtained regression coefficients (*r^2^)* indicate whether or not a correlation exists between the motion and the minimum distance between the stomach and the LVCs.

## 3 RESULTS

### 3.1 Relative COM translations

No statistical difference was found for all cardiac structures for magnitude COM translations between FB and AC patients (med. FB: 8.3 mm, AC: 7.7 mm; *p* > 0.05). Table 3 compares FB and AC cardiac structure magnitude COM translation for the total patient dataset and the three VT patients. The use of AC to reduce cardiac motion varied between the three VT patients. AC reduced magnitude COM translations for all cardiac structures on VT Patient 1 by an average of 3.3 mm, reduced motion in two out of seven cardiac structures on VT Patient 2 by an average of 1.1 mm, and reduced motion for four out of seven cardiac structures for VT Patient 3 by an average of 3.6 mm. AC increased magnitude COM translation for five out of seven cardiac structures on VT Patient 2 by an average of 1.7 mm, and increased motion for three out of seven cardiac structures on VT Patient 3 by an average of 2.2 mm.

**TABLE 3:** Magnitude COM translations for delineated cardiac structures between FB and AC datasets.

	Magnitude COM Translation [mm] FB, AC				
Cardiac structures	Median (*N* = 42)^a^	*p*	VT Patient 1	VT Patient 2	VT Patient 3
Heart	**6.6**, 6.3	0.30	**6.6,** 4.0	6.3, **8.0**	4.8, **5.0**
LV	**7.0**, 6.6	0.72	**6.2,** 4.9	7.0, **7.8**	**5.0**, 4.5
LVC—anterior	**8.9**, 8.4	0.86	**11.4,** 4.6	**9.6,** 8.5	6.5, **9.1**
LVC—apical	**9.5**, 7.4	>0.05	**11.1,** 8.8	8.6, **8.7**	**13.6**, 5.7
LVC—inferior	8.5, **9.3**	0.21	**8.8,** 6.3	**12.6,** 11.5	5.9, **9.7**
LVC—lateral	**8.7**, 7.9	0.33	**10.5,** 7.5	6.7, **10.6**	**13.0,** 7.9
LVC—septal	**7.8**, 7.2	0.21	**10.6,** 6.3	8.1, **10.0**	**7.7**, 7.0

*Note*: Magnitude COM translation for delineated cardiac structures between the total FB and AC datasets (*N* = 42) and the three VT patients. Shown in **bold** are the greater of the two COM translations.

^a^ Total patient dataset consisting of lung (*n* = 18), liver (*n* = 18), and VT patients (*n* = 3); shown in **bold** are the greater of the two COM translations.

Table 4 shows the magnitude COM translation and ROM of the stomach for the total patient dataset and the three VT Patients. There was no statistical difference between FB and AC stomach ROM in the LR (med. FB: 3.0 mm, med. AC: 2.1 mm; *p* = 0.24) and SI (med. FB: 7.7 mm, med. AC: 7.4 mm; *p* = 0.24) direction or magnitude COM translation; however, there was a statistically significant difference in the AP ROM (med. FB: 4.5 mm, med AC: 2.6 mm; *p* = 0.01) due to the application of AC in the AP direction. The effect of AC on the magnitude of stomach COM translation was variable for the three VT patients: AC reduced stomach motion for VT Patients 1 and 2 but increased stomach motion for VT Patient 3. Stomach ROM under AC was reduced in all directions for VT Patient 1, reduced in the AP and SI, but increased in the LR for VT Patient 2, and increased in all directions for VT Patient 3.

**TABLE 4:** Stomach COM translation between FB and AC datasets.

	Median (*N *= 42)^a^		VT Patient 1	VT Patient 2	VT Patient 3
Stomach	FB, AC	*p*	FB, AC	FB, AC	FB, AC
Magnitude COM Translation [mm]	**8.6**, 8.1	*0.46*	**19.8**, 7.3	**8.6**, 5.9	6.4**, 10.3**
ROM					
LR	**3.0**, 2.1	*0.24*	**8.9**, 2.1	3.1, **3.7**	4.9, **5.2**
AP	**4.4**, 2.7	*0.01*	**13.7**, 2.8	**7.2**, 2.7	1.6, **4.3**
SI [mm]	**7.7**, 7.4	*0.76*	**11.5**, 7.0	**7.3**, 5.2	5.3, **9.1**

*Note*: Stomach COM translation in magnitude and ROM in the LR, AP, and SI direction for FB and AC datasets and the three VT patients. Shown in **bold** are the greater of the two COM translations.

^a^ Total patient dataset consisting of lung (*n* = 18), liver (*n* = 18), and VT patients (*n* = 3).

**FIGURE 1:**
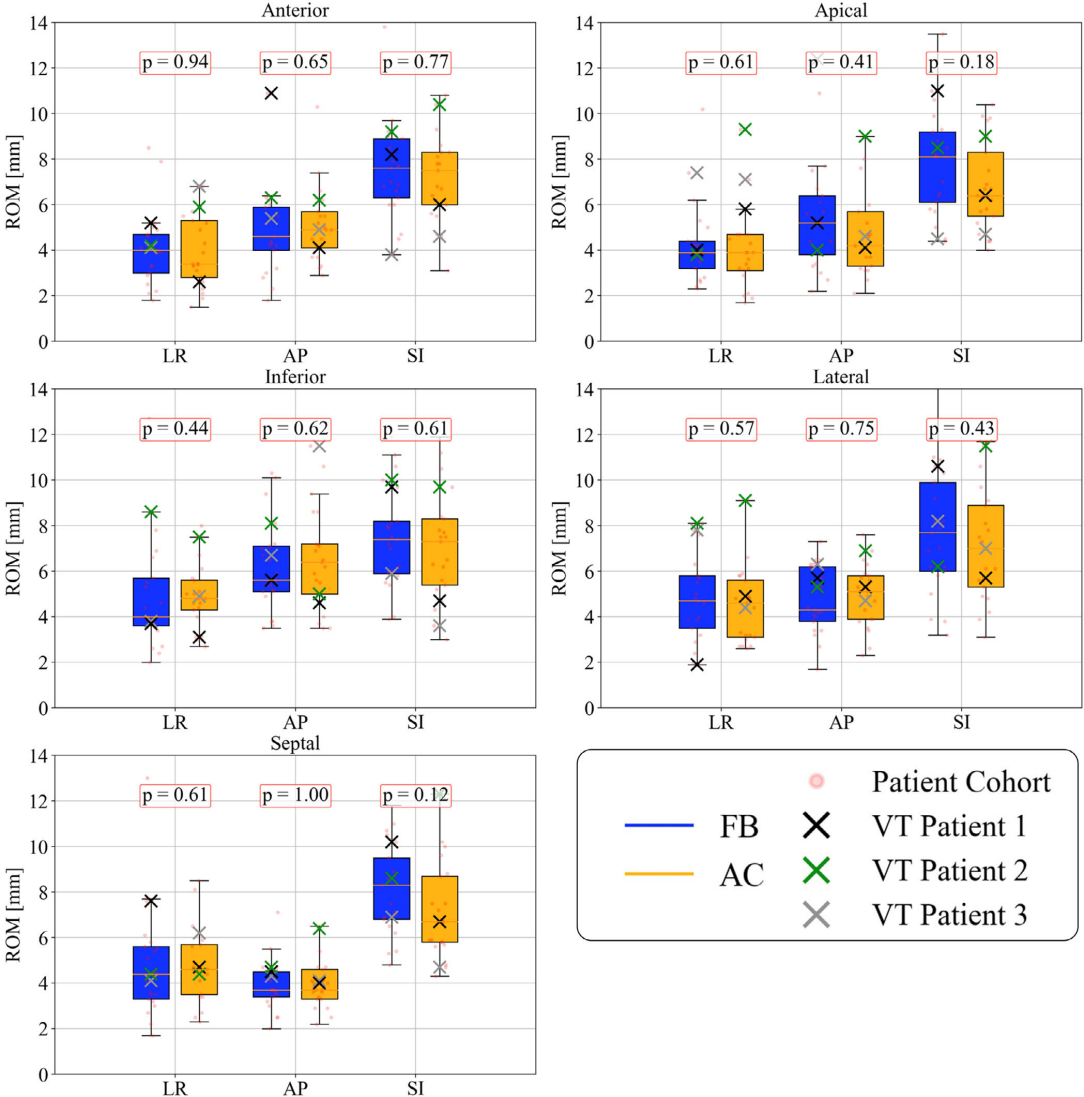
ROM of the five LVCs in the LR, AP, and SI directions. ROM in the LR, AP, and SI direction for the five LVCs for both FB and AC over the total patient dataset. *p*‐values with significance *α* = 0.05 are shown in the red boxes.

No statistical difference was observed between FB and AC ROM data for all LVCs shown in Figure 1 (total dataset; *p* > 0.17). For the three VT patients, ROM reduction under AC was LVC and direction‐specific for all patients (Table 5). Applying simplified anisotropic ITV margins from the ROM analysis to the GTVs of the three VT patients demonstrates treatment volume reduction under AC is also patient‐specific (Table 6): AC reduced ITV volumes for VT patients 1 and 3 by 24% and increased ITV volume for VT Patient 2 by 24% compared to FB.

**TABLE 5:** LVC ROM delta between FB and AC for the three VT patients.

ROM Δ = AC‐FB [mm]	LR P1, P2, P3	AP P1, P2, P3	SI P1, P2, P3
Anterior	−2.6, 1.8, 2.6	−6.8, −0.2, −0.4	−1.8, 1.2, 0.8
Apical	1.8, 5.6, −0.2	−1.0, 5.0, −7.8	−4.6, 0.4, 0.2
Inferior	1.2, −1.0, 1.2	1.8, −3.2, 4.8	−0.6, −0.2, −2.4
Lateral	3.0, 1.0, −3.4	−0.4, 1.2, −1.6	−4.8, 5.4, −1.2
Septal	−2.8, 0.0, 2.0	−0.6, 1.6, −0.2	−3.4, 3.8, −2.2

*Note*: ROM Δ is the difference in AC and FB COM translation in the LR, AP, and SI directions. A negative number indicates reduced motion under AC.

**TABLE 6:** Anisotropic ITV margins and treatment volumes generated from ROM analysis on VT patients.

	VT Patient 1	VT Patient 2	VT Patient 3
ITV [mm]			
LR, AP, SI			
FB	4, 5, 5	4, 3, 4	4, 3, 4
AC	2, 3, 5	5, 4, 6	3, 2, 3
ITV [cc]			
FB	71.3	120.4	39.0
AC	55.3	149.0	29.8
*AC/FB*	*0.76*	*1.24*	*0.76*

*Note*: Anisotropic ITV margins are applied to the respective GTVs of the three VT patients.

### 3.2 Relative distance between LVCs to stomach

Minimum distances between the LVCs and the stomach over all 4DCT phases can be found in Table 7 for the total patient dataset (*N* = 42) and for the three VT patients. Over the total dataset, there was no statistical difference between FB and AC patients for the minimum distances between LVCs and the stomach over the 4DCTs (*p* > 0.14). For the anterior and septal LVCs, distances to the stomach were never within the defined 3 or 5 mm margins. The apical, inferior, and lateral LVCs were more likely to fall within these margins, which could result in stomach‐treatment volume overlap. For these latter LVCs, the median of the minimum distances to the stomach were 2.1 and 3.3 mm for the apical, 6.8 and 8.7 mm for the inferior, and 3.0 and 4.5 mm for the lateral LVC for FB and AC, respectively. For at least one phase, more than 61%, 19%, and 52% of all patients (FB and AC) fell within a 3 mm margin for the apical, inferior, and lateral LVCs, respectively, and greater than 76%, 33%, and 71% of all patients fell within a 5 mm margin for the apical, inferior, and lateral LVCs, respectively. Note that with larger treatment margins (>5 mm), the frequency at which a patient's stomach is found to fall within a set distance to the target would likely increase.

**TABLE 7:** Minimum distances between LVCs and stomach for FB and AC datasets.

	Minimum distance [mm] FB, AC			
LVCs	Full dataset (*N *= 42^a^) Median *p*	VT Patient 1	VT Patient 2	VT Patient 3
Anterior	38.3, 38.7 *0.69*	30.0, 31.9	29.7, 40.0	46.7, 50.7
Apical	**2.1, 3.3** *0.44*	**2.1, 4.0**	**2.0, 2.0**	**4.7**, 10.9
Inferior	6.8, 8.7 *0.42*	6.7, 9.9	8.0, 6.0	10.7, 20.1
Lateral	**3.0, 4.5** *0.44*	7.9, 9.3	**4.0, 4.0**	**4.8** , 10.6
Septal	20.1, 23.4 *0.14*	24.6, 26.0	14.1, 16.8	34.4, 38.5

*Note*: Minimum distances from the LVCs to the stomach between FB and AC for the total patient dataset (*N* = 42) and the three VT patients. Shown in **bold** are distances within a 5 mm margin to the stomach. Underlined values for the three VT patients correspond with the LVC location of the respective GTVs.

^a^ Total patient dataset consisting of lung (*n* = 18), liver (*n* = 18), and VT patients (*n* = 3).

The impact of AC on the minimum distances from the LVCs to the stomach was non‐uniform between the three VT patients. For VT Patients 1 and 2, AC did not increase distances between the LVCs and the stomach past the 5 mm margin. However, for VT Patient 3, AC increased the distance between the stomach to the apical and lateral component past the 5 mm margin, indicating that AC did benefit this patient by reducing the possibility of stomach‐treatment volume overlap.

**FIGURE 2:**
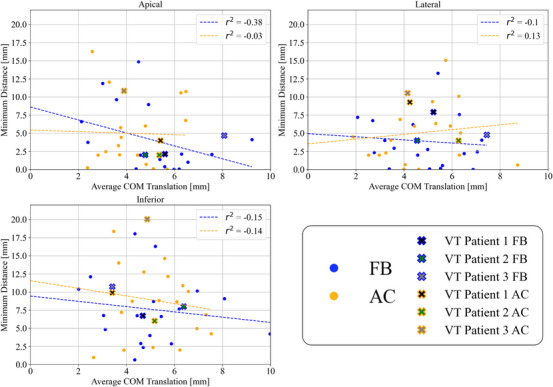
Correlation between translation and separation distance to stomach for the apical, lateral, and inferior LVC. Linear regression analysis of the separation distance between the apical, inferior, and lateral LVC and stomach, and average magnitude COM translation over the respiratory‐cycle of the respective LVC.

Figure 2 shows the linear regression analysis correlating averaged magnitude COM translation over the respiratory‐cycle of the apical, inferior, and lateral LVCs (as these were the only LVCs found to fall within the 3 or 5 mm margin) to the minimum distance from the respective LVC to the stomach over the respiratory‐cycle for both FB and AC. No correlation (*r^2^* ∈ [−0.38–0.13]) is observed between the two variables for all three LVCs and for either FB or AC. AC did not increase the correlation between the two variables. These results imply that the amount of magnitude COM translation of the LVCs did not elicit a change in the possibility of stomach‐treatment volume overlap.

## 4 DISCUSSION

No significant differences were found in magnitude motion between FB and AC patient datasets for the contoured cardiac structures. In addition, a magnitude motion reduction under AC for the three STAR patients was shown to be patient‐dependent. These results are consistent with those presented in a lung tumor study by Rasheed et al.^30^ who compared lung tumor and whole heart motion of patients imaged FB and under AC. The authors showed that AC reduced heart motion in 10 patients (average of 2.1 mm) but increased heart motion in the remaining seven (average of 1.3 mm); they remarked that AC produces a patient‐specific effect on heart motion. The results herein for the heart, LV, and LVCs showed the same effect. To our knowledge, our study is the first to examine the differences in the magnitude motion of the heart and cardiac substructures between FB and AC.

When evaluating the directional motion of cardiac structures (ROM), it was found that the impact of AC compared to FB in the LR, AP, and SI direction was insignificant for all LVCs for the total patient dataset. For the three VT patients, the observed effect of AC on their respective ROMs demonstrates a non‐uniform and inconsistent effect between patients, direction, and target location. For some patients, a reduction in motion of a cardiac structure under AC compared to FB was observed, however, this structure also exhibited increased motion under AC in one of the three cardinal directions. This effect was observed for all three VT patients. For example, VT Patient 2 had a reduction in magnitude motion of 1.1 mm for the anterior LVC, whereas the anterior LVC increased in LR ROM by 1.7 mm. Therefore, a reduction in the magnitude motion of a structure under AC compared to FB does not directly correlate with reduced ROM in all directions or a reduced final treatment volume.

Consistent with results presented in previous studies,^6^, ^14^ the relative magnitude of SI motion compared to AP and LR motion for the five LVCs demonstrates that anisotropic ITV margins may best encapsulate target motion variability over the respiratory cycle. However, as discussed by Brandner et al.^31^ and Dvorak et al.,^6^ the relative difference in normal tissue exposure between anisotropic treatment margins that more closely encapsulate variable target motion and isotropic ITV margin expansions should be evaluated. The calculated ITV margins demonstrate that AC has a patient‐specific effect on both the direction of target motion and magnitude of motion and thus, the resulting volume of the required ITV. The method of taking the largest ROM between LVCs to generate the ITV resulted in the largest required margin to encapsulate the target motion. Therefore, this method likely generated larger ITVs than needed for the actual exhibited target motion, as seen in the difference between the calculated ITVs and the clinically applied ITVs in Table 2. Despite this, the analysis is useful to compare treatment volumes between FB and AC.

Another unknown consideration for this treatment technique is the requirement for target coverage to result in a reduction of VT episodes. Contrary to treatments of malignant diseases where irradiation of each cell is essential to achieve local control, this may not be necessary for STAR treatments. In addition, the target definition is not yet well understood, and the uncertainties in defining the scar location are larger and not well quantified. The isotropic 2 mm ITV margins applied for two of the three VT patients are smaller than those presented in Table 6; however, limiting toxicity takes precedence over using larger ITV margins that can guarantee all motion is fully captured.

The effect of AC on stomach magnitude COM translation was not statistically significant over the total patient dataset and was variable for the three VT patients. While AC shows some reduction in the stomach AP ROM, it did not affect the relative distance from the stomach to the LVCs and did not demonstrate a consistent clinical advantage for STAR patients.

Stomach overlap risk with the 3‐ and 5‐mm margins was comparable between FB and AC patients for all five LVCs. Expected from their anatomical locations, the apical, inferior, and lateral LVCs presented a higher possibility of stomach‐treatment margin overlap than the anterior and septal LVCs. The effect of AC on the minimum distance between the apical, inferior, and lateral LVC and the stomach for VT Patients 1 and 2 was not clinically significant. Conversely, VT Patient 3 demonstrated an increase in minimum distance between the apical and lateral LVCs and the stomach past the 5 mm margin under AC, indicating that AC potentially reduced stomach‐treatment volume overlap for this patient. However, as mentioned earlier, Gerard et al.^18^ observed the stomach closer to the PTV under AC, making both outcomes possible. If AC is employed as a STAR target motion management method, the impact of AC on the relative distance between the stomach and the target volume should be carefully evaluated on a patient‐to‐patient basis.

Certain patients may demonstrate reduced respiratory‐induced motion of the LVCs or increased separation distance from the target to the stomach with AC; however, it is currently unknown what patient characteristics could indicate this. The variability observed in breathing patterns and the effect of AC across the patients in our study may be attributed to a few elements: differences in lung volume, the available compression level based on the patient's body mass index and their tolerance of the compression, the way the patient is breathing (abdominal vs. chest breathing). The aforementioned patient characteristics may have contributed to the observed patient variability in this study. As described by Stevens et al.,^23^ the limited available information on cardiac‐respiratory motion has led to difficulty in prescribing the optimal motion‐compensation methods. Based on the authors’ conclusions and the results presented in this study, further research is recommended to evaluate the effect of the aforementioned patient characteristics on cardiac‐respiratory motion and the optimal motion‐management technique for STAR.

In an effort to minimize the uncomfortable immobilization experience and reduce the occurrence of two 4DCT scans with and without AC performed on each patient, recommendations are provided, considering the time, resources, and additional radiation exposure involved. It is recommended patients first undergo a FB 4DCT scan with contrast. A second 4DCT scan with AC following the FB scan (in the same CT session) should only be performed if the FB 4DCT scan shows significant motion in the heart/LV or stomach (≳5 mm) or if the target volume is within the apical, inferior, or lateral LVC. It should be noted that if significant motion or proximity of the stomach to the target volume is observed on the FB 4DCT, there is no guarantee that the second 4DCT with AC will reduce motion or reduce the likelihood of stomach treatment volume overlap. Regarding the VT patients within this study, based on their measured target motion, VT Patients 1 and 3 would have benefited from the additional AC 4DCT. The recommended instances for performing the AC 4DCT scan are where the highest potential for patient benefit lies.

One limitation of the current study is the existing patient dataset containing only three STAR patients imaged under both FB and AC conditions. Direct structure motion and minimum distance comparison between FB and AC are, therefore, limited to the three treated VT patients. Including a larger dataset of STAR patients imaged with and without AC would enable direct target‐specific motion comparisons between FB and AC. However, results from the STAR patient cohort were consistent with those from the total patient cohort of lung and liver SBRT patients and agreed with previously published results.^14^, ^30^ A recent review by Stevens et al.^23^ evaluated cardiac motion in multiple ventricular radioablation studies. The review reported on average, cardiac‐respiratory target motion of subjects with VT was <5 mm, with motion extending in the Sup/Inf, Ant/Post, and Left/Right direction 8.0, 5.2, and 6.0 mm, respectively. The median reported motion in our study is <6 mm in the LR and AP direction and <9 mm in the SI direction when considering the total patient dataset of lung, liver, and VT patients. The consistency between the motion reported from the current dataset and that which is reported in the literature for VT patients indicates the dataset in this study is sufficient for the given analysis. The target motion of the included VT patients in the study was <6 mm in the LR, AP, and SI direction, and were all within ±2 σ of the median of the total patient dataset. A second limitation of the study is the comparison of FB and AC structure translations being limited to the COM analysis. A COM analysis does not consider structural variations, such as rotations around different axes or volume and shape changes. Including these structural variations would enable a more precise definition of ITV margins under FB and AC for STAR patients. Last, thoracic respiratory‐gated 4DCTs have inherent cardiac‐cycle motion captured in the respiratory phases. This cardiac motion cannot be effectively sampled and evaluated from a respiratory‐gated 4DCT alone. As discussed by Prusator et al.,^14^ VT patient target motion from the cardiac‐respiratory cycle is comparable to target motion from only the cardiac cycle, implying motion captured of the target volume under a respiratory‐gated 4DCT is still adequate to capture the motion extent of the target volume over the cardiac‐respiratory cycle. Despite these limitations, the results presented herein still directly compare FB and AC of STAR target ROM as the same methodology was applied to all patients. Additionally, improving ITV definition accuracy may not be required, as briefly discussed above, and as evidenced by published results of STAR patients treated with smaller ITV margins (or no ITV margin) that reported significant VT burden reduction.^1^, ^8^, ^27^, ^28^, ^29^ It is not clear at this time the exact target tissue required to result in a clinical improvement.

## 5 CONCLUSION

The results of this study indicate that AC as a respiratory‐motion management device did not benefit all STAR patients. AC did not uniformly or consistently reduce cardiac structure motion throughout the respiratory cycle for each patient, and it did not lessen the possibility of stomach overlap with the treatment volume during the respiratory cycle. AC could be employed if significant target motion is observed on a FB scan which would otherwise affect target coverage due to the proximity of the stomach to the target volume.

## AUTHOR CONTRIBUTIONS

Daniel Cecchi was responsible for all data collection and analysis in this document and for procuring a temporary license to use Limbus AI for autocontouring. Dr. Hali Morrison obtained ethics approval to use patient images. All listed authors contributed to the document's writing, revisions, and completion.

## CONFLICT OF INTEREST STATEMENT

The authors have no conflicts of interest to report.

## Data Availability

The data supporting this study's findings are available from the corresponding author upon reasonable request.
